# A proposal for T1 subclassification in hepatocellular carcinoma: reappraisal of the AJCC 8th edition

**DOI:** 10.1007/s12072-022-10422-8

**Published:** 2022-09-28

**Authors:** Chao-Wei Lee, Hsin-I Tsai, Ming-Chin Yu, Chih-Chi Wang, Wei-Chen Lee, Ta-Sen Yeh, Chun-Nan Yeh, Cheng-Yu Lin, Tony Kuo, Hsing-Yu Chen

**Affiliations:** 1 Division of General Surgery, Department of Surgery, Linkou Chang Gung Memorial Hospital, Guishan, Taoyuan, Taiwan; 2 College of Medicine, Chang Gung University, Guishan, Taoyuan, Taiwan; 3 Graduate Institute of Clinical Medical Sciences, Chang Gung University, Guishan, Taoyuan, Taiwan; 4 Department of Anesthesiology, Linkou Chang Gung Memorial Hospital, Guishan, Taoyuan, Taiwan; 5 Department of Surgery, New Taipei Municipal Tu-Cheng Hospital (Built and Operated By Chang Gung Medical Foundation), Tu-Cheng, New Taipei City, Taiwan; 6 Division of General Surgery, Department of Surgery, Kaohsiung Chang Gung Memorial Hospital, Kaohsiung, Taiwan; 7 Division of General Surgery, Department of Surgery, Chiayi Chang Gung Memorial Hospital, Chiayi, Taiwan; 8 Department of Gastroenterology and Hepatology, Linkou Chang Gung Memorial Hospital, Guishan, Taoyuan, Taiwan; 9 Division of Chinese Internal Medicine, Center for Traditional Chinese Medicine, Taoyuan Chang Gung Memorial Hospital, No.123, Dinghu Rd., Guishan Dist., Taoyuan, 33305 Taiwan; 10 School of Traditional Chinese Medicine, College of Medicine, Chang Gung University, Guishan, Taoyuan, Taiwan

**Keywords:** Hepatocellular carcinoma, 8th edition of American Joint Committee on Cancer (AJCC), T1 subclassification, Liver resection, Chang Gung Research Database

## Abstract

**Background:**

In the 8th edition of American Joint Committee on Cancer (AJCC) staging system for hepatocellular carcinoma (HCC), tumor size is not considered in T1 stage. The present study aimed to find out the optimal cutoff for tumor size to further stratify patients with T1 HCC.

**Methods:**

Operated HCC patients were identified from the Chang Gung Research Database (CGRD), and the patients with T1bN0M0 tumors were further divided into two groups based on the tumor size. The resulting subgroups were denoted as T1b (≤ cutoff) and T1c (> cutoff). The survivals were compared between T1a/b and T1c as well as T1c and T2.

**Results:**

From 2002 to 2018, a total of 2893 patients who underwent surgery for T1N0M0 HCC were identified from the CGRD. After excluding cases who died within 30 days of surgery, Kaplan–Meier survival analysis discovered that T1 tumors > 65 mm (T1c) had survivals similar to those of T2N0M0 tumors. Cox regression multivariate analysis further demonstrated that tumor size > 6.5 cm was an independent poor prognostic indicator for T1 HCC. Sensitivity tests also confirmed that tumors lager than 6.5 cm were significantly more likely to develop both tumor recurrence and liver-specific death after surgery.

**Conclusions:**

Our study demonstrated that tumor size would significantly impact the survival outcome of T1 HCC after surgery. Due to significantly worse survival, we proposed a subclassification within T1 HCC, T1c: solitary tumor > 6.5 cm without vascular invasion, to further stratify those patients at risk. Further studies are mandatory to validate our findings.

**Supplementary Information:**

The online version contains supplementary material available at 10.1007/s12072-022-10422-8.

## Introduction

Hepatocellular carcinoma (HCC) is the most common primary malignancy of the liver and 6th most common cancer worldwide, with an estimated death of approximately 830,000 worldwide in 2020 [1–4]. In Taiwan, it is the fourth most common cause of cancer death and causes more than 7000 deaths each year [5]. Surgical resection, radiofrequency ablation, and liver transplantation remain the most effective curative therapies in selected patients. However, unlike other solid malignancies, the treatment of HCC must take multiple important factors into considerations. For example, the coexisting underlying liver diseases, such as chronic hepatitis B or C and alcoholic liver disease, had limited the extent and feasibility of liver resection. According to a recent analysis, only around 5–40% of non-cirrhotic HCC patients underwent liver resection, and this percentage was even much lower if the patients have chronic liver disease or overt cirrhosis [6].

Due to heterogeneous disease presentation and poor prognosis, many staging systems thus have been proposed to suggest appropriate treatment and predict survival outcome for patients with HCC. Among them, the American Joint Committee on Cancer (AJCC) Tumor/Node/Metastasis (TNM) staging system is one of the most commonly used staging systems to stratify the prognosis of patients with HCC [7, 8]. The latest edition, 8th edition, was released in December 2016 to further optimize the prognostic capability of the 7th edition [9, 10]. Major vascular invasion, for example, has been upgraded from T3b to T4 in this version. Despite important modifications, however, the newest edition has not stratified the tumors beyond 2 cm based on size. The staging and prognosis of either solitary small or large HCC without vascular invasion, based on this system, are essentially the same. Nevertheless, there were many other studies demonstrating that tumor diameter, in addition to vascular invasion, was also an important prognostic factor for HCC [11–14]. Another recent study even showed that there was an apparent survival difference among stage I HCC patients with different tumor sizes [15]. Patients with larger HCC had significantly higher risks of tumor recurrence and death than those with smaller tumors after liver resection [15]. As a result, it is of urgent needs to reassess the impact of tumor diameter on the outcome of HCC and to enhance the staging system. The current study, by utilizing the data from the Chang Gung Research Database (CGRD), aimed to find out the optimal cutoff for tumor size to further stratify the 8^th^ edition of AJCC TNM staging system [15–18]. To eliminate the potential bias due to different stages and treatment approaches, we examined patients with pathologically proven T1N0M0 HCC who underwent curative intent liver resection.

## Materials and methods

### Data source

The CGRD, which collected the clinical information from eight Chang Gung memorial hospitals (CGMH) in Taiwan since year 2000, was the primary data source of the current research. With more than 10,070 beds and 500,000 emergency visits each year, the CGRD has accounted for 21.2% of outpatients and 12.4% of inpatients in Taiwan and become an excellent database for various kinds of clinical studies [15–18]. For cancer patients, it contains comprehensive cancer registry maintained in a prospective manner. The information is manually validated with a high completeness rate [19, 20]. Both the International Classification of Diseases, 9th and 10th revision, Clinical Modification (ICD-9-CM and ICD-10-CM) codes and the International Classification of Diseases for Oncology, 3rd edition (ICD-O-3) are used in the CGRD. For personal privacy, the individual identity is protected by encryption. The medical information is prospectively digitalized and stored in the CGRD and is amenable for researchers to perform large-scale retrospective analysis.

### Study design and population

**Fig. 1:**
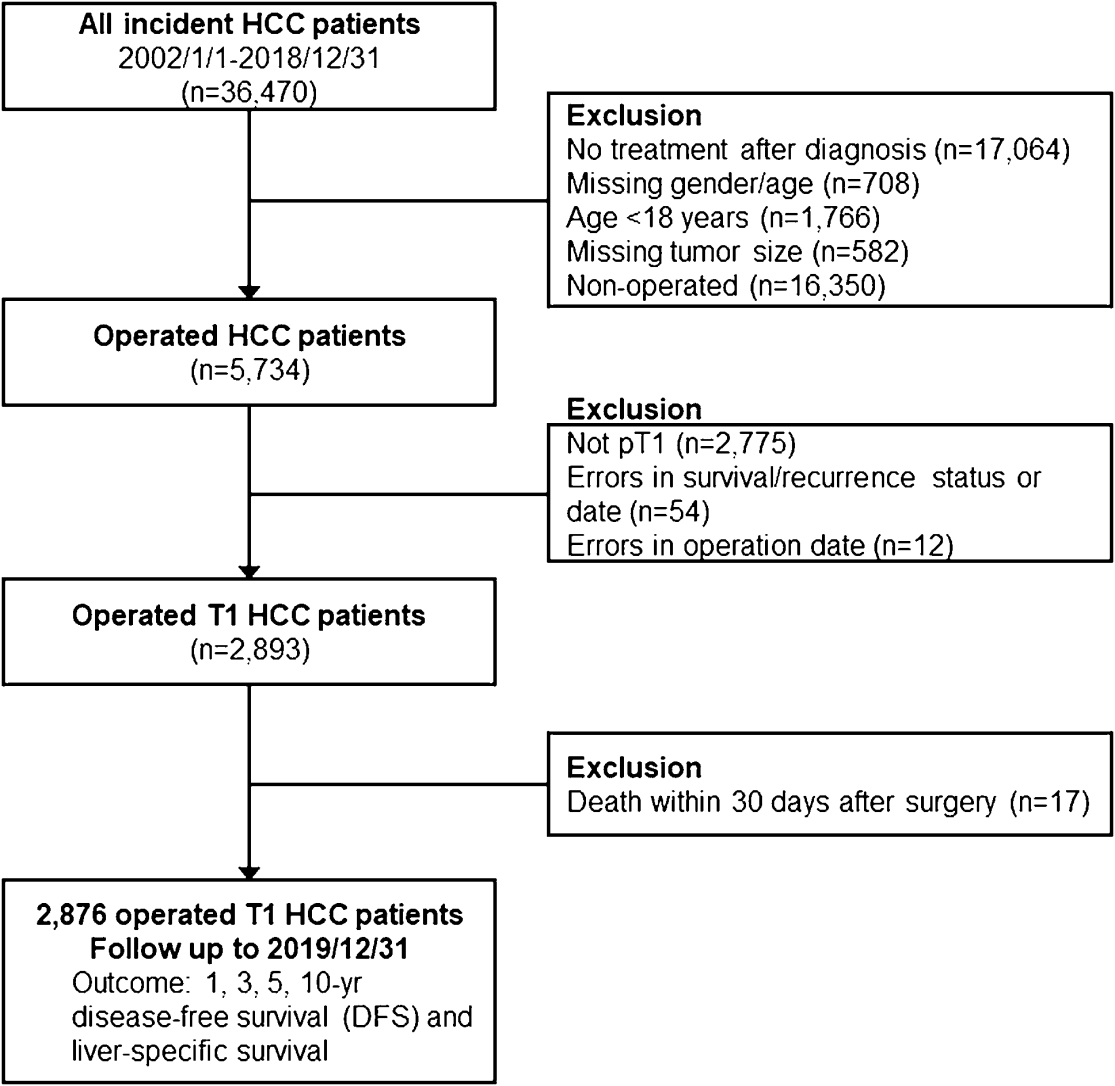
Flow diagram of the current study. HCC patients diagnosed from 2002 to 2018 were retrieved from the CGRD database (*n* = 36,740). Those who received non-surgical treatment, who had missing data, or who died within 30 days of surgery were excluded from further analysis. The patients with T1bN0M0 tumors were further divided into two groups based on the tumor size, and the cutoff value was set from 3 cm and increased by a 1-cm increment. The disease-free survival (DFS) as well as liver-specific overall survival (OS) were analyzed and compared

Figure 1 shows the flow diagram of the current study. The ICD-9-CM code 1550 and ICD-10-CM code C220 were employed to identify HCC patients from the CGRD. Patients who received curative operation from 2002 to 2018 were enrolled as the study population. Those who received non-surgical treatment, who had missing data, or who died within 30 days of surgery were excluded from further analysis. Tumors were staged according to the 8th edition of AJCC TNM staging system in the current study [9, 10]. To explore the optimal cutoff for tumor size, a total of 2876 pathologically proven pT1N0M0 HCC were further identified from the operated cohort.

The patients with T1b tumors were then divided into two groups based on the tumor size, and the cutoff value was set from 3 cm and increased by a 1-cm increment. The resulting subgroups were denoted as T1b (≤ cutoff) and T1c (> cutoff). Kaplan–Meier survival curves were acquired and disease-free survival (DFS) as well as liver-specific overall survival (OS) were analyzed and compared between T1a/b and T1c by log-rank tests. The survivals between T1c and T2N0M0 tumors were also compared. The smallest cutoff with comparable survival outcome between T1c and T2 tumors was designated as the optimal cutoff value. This study was approved by the Institutional Review Boards of CGMH (IRB No.: 202000608B0).

### Outcome assessment and statistical analysis

DFS and liver-specific OS were used as the primary and secondary outcomes of the current study, respectively. The first date of definite diagnosis for HCC was set as the index date. DFS defined the period between the index date and the date of the first documented clinical recurrence or the end of year 2019. Liver-specific OS spanned the period between the index date and the date of liver-cause mortality or the end of year 2019. The liver-causes included tumor recurrence, metastasis, and complications of decompensated liver cirrhosis.

Kaplan–Meier survival estimation with log-rank test was used to assess the DFS and liver-specific OS. Cox regression multivariate analysis was performed to identify significant prognostic factors associated with disease recurrence or liver-cause mortality for T1 HCC. Five different Cox regression models incorporating multiple prognostic factors were also established as the sensitivity tests to assess the consistency of incremental risks associated with the new subclassification of T1 HCC. The freeware Konstanz information miner (KNIME) and the commercial statistic software STATA (StataCorp. 2019. Stata Statistical Software: release 16. College Station, TX: StataCorp LLC) were employed to process and analyze the data [21]. All statistics with *p* < 0.05 were regarded as statistically significant.

## Results

### Patient demographics

We first identified 36,470 patients diagnosed to have HCC from the CGRD. Among them, 5734 patients were operated by liver resection for their HCC. After excluding patients who had more than T1 disease (*n* = 2775), who had erroneous clinical information (*n* = 66), and who died within 30 days of surgery (*n* = 17), a total of 2876 patients were enrolled into our final analysis (Fig. 1). Among these patients who had T1N0M0 HCC, 2172 (75.5%) were male and 1430 (49.7%) were older than 60 years. Hepatitis B virus (HBV) infection remained the most common etiology (48.2%), followed by hepatitis C virus (HCV) infection (24.6%). While nearly half of the patients operated had histologically proven liver cirrhosis, they were mostly (nearly 99%) Child classification A. The mean tumor size was 35.9 mm and the mean alpha-fetoprotein was 2471.8 ng/mL. Nearly 70% of the patients enrolled were categorized as albumin–bilirubin (ALBI) grade 1 and about 75% were either normally nourished or mildly malnourished (Table 1).

**Table 1:** Baseline features of T1 hepatocellular carcinoma patients undergoing liver resection (AJCC 8th version), *n* (%), *n* = 2876

Gender	
Female	704 (24.5%)
Male	2172 (75.5%)
Age [mean (SD)] (year)	59.0 (11.2)
Age group	
− 20	2 (0.1%)
21–40	210 (7.3%)
41–60	1234 (42.9%)
61–	1430 (49.7%)
Co-morbidities	
Diabetes	717 (24.9%)
Hypertension	1005 (34.9%)
Chronic hepatitis	
HBV	1385 (48.2%)
HCV	708 (24.6%)
HBV+HCV	142 (4.9%)
Lifestyles	
Cigarette smoking	298 (10.4%)
Alcohol consumption	255 (8.9%)
Betel nut	79 (2.7%)
Child–Turcot–Pugh classification	
A	1464 (98.9%)
B	16 (1.1%)
Cirrhosis	
No	778 (51.0%)
Yes	748 (49.0%)
Tumor size [mean (SD)] (mm)	35.9 (26.7)
Medications	
Anti-HCV/HBV therapy	279 (9.7%)
Metformin	186 (6.5%)
Aspirin	126 (4.4%)

Biochemical profiles	*n* (%),* n* = 2876^1^
Alpha-fetoprotein [mean (SD)] (ng/mL)	2471.8 (56,299.0)
ICG-15 [mean (SD)] (%)	9.4 (8.3)
Albumin [mean (SD)] (g/dL)	4.1 (0.5)
Hemoglobin [mean SD)] (g/dL)	13.6 (1.9)
Platelet [mean (SD)] (1000/μL)	175.0 (68.8)
INR [mean (SD)]	1.1 (0.1)
AST (U/L) [mean (SD)]	57.4 (97.2)
ALT (U/L) [mean (SD)]	61.9 (98.7)
Total bilirubin (mg/dL) [mean(SD)]	0.9 (0.6)
ALBI grade	
Grade 1	1629 (69.8%)
Grade 2	676 (29.0%)
Grade 3	27 (1.2%)
PNI	
Normal	1005 (49.0%)
Mild malnutrition	531 (25.9%)
Moderate to severe malnutrition	325 (15.9%)
Serious malnutrition	189 (9.2%)
NLR [mean (SD)]	4.0 (5.4)
PLR [mean (SD)]	17.3 (6.9)

^1^Number excluded surgical mortality (30-day mortality)

*ALBI* albumin–bilirubin grade, *ALT* alanine aminotransferase, *AJCC* American Joint Committee on Cancer, *AST* aspartate aminotransferase, *HBV* hepatitis B virus, *HCV* hepatitis C virus, *ICG-15* indocyanine green retention test at 15 min, *INR* international normalized ratio, *NLR* neutrophil-to-lymphocyte ratio, *PLR* platelet-to-lymphocyte ratio, *PNI* prognostic nutritional index, *SD* standard deviation

### Subclassification of T1 HCC

**Fig. 2:**
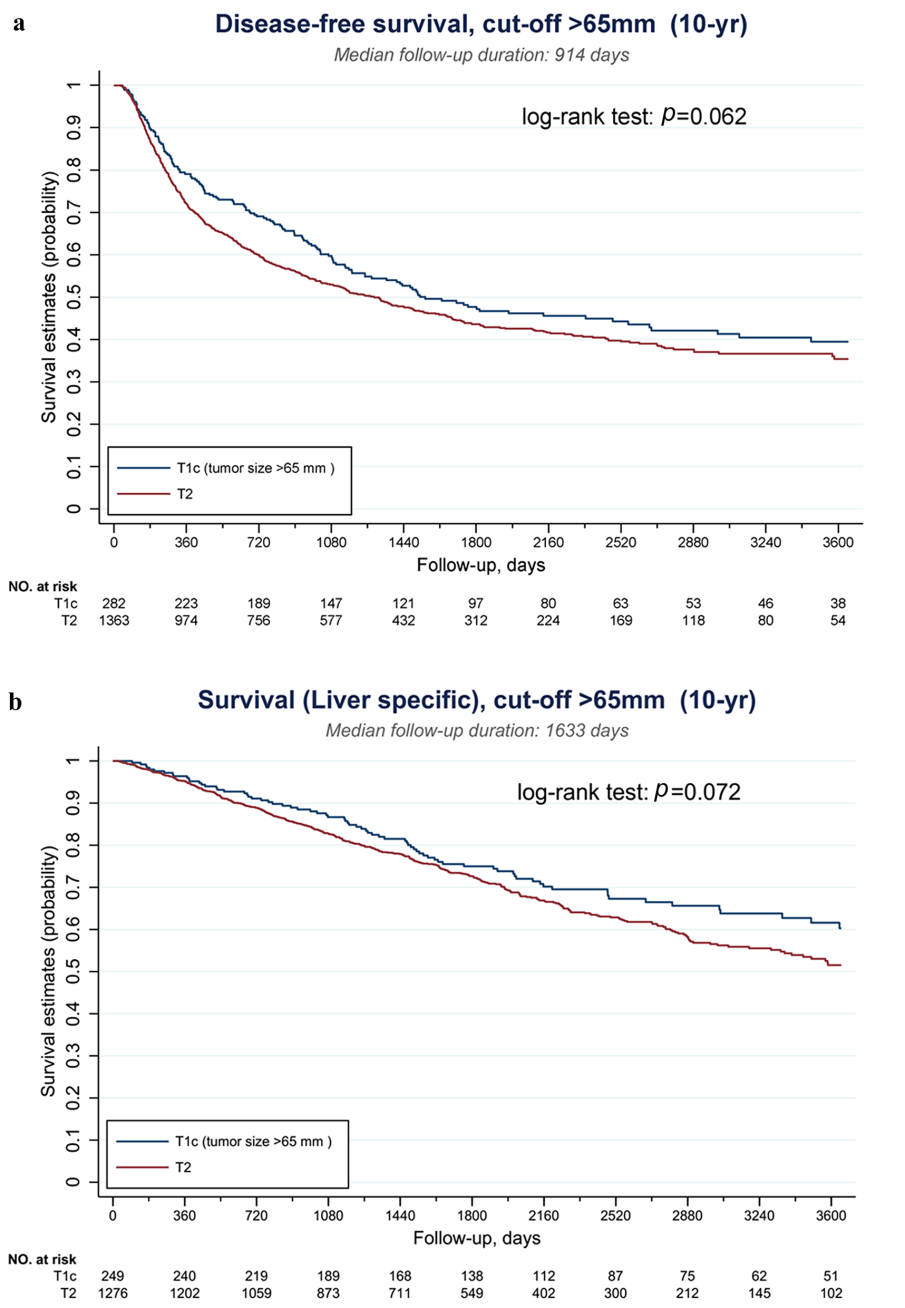
Survivals of solitary HCC > 6.5 cm were not statistically different from those of T2 tumors. **a** Disease-free survival (DFS) and **b** liver-specific overall survival (OS)

**Fig. 3:**
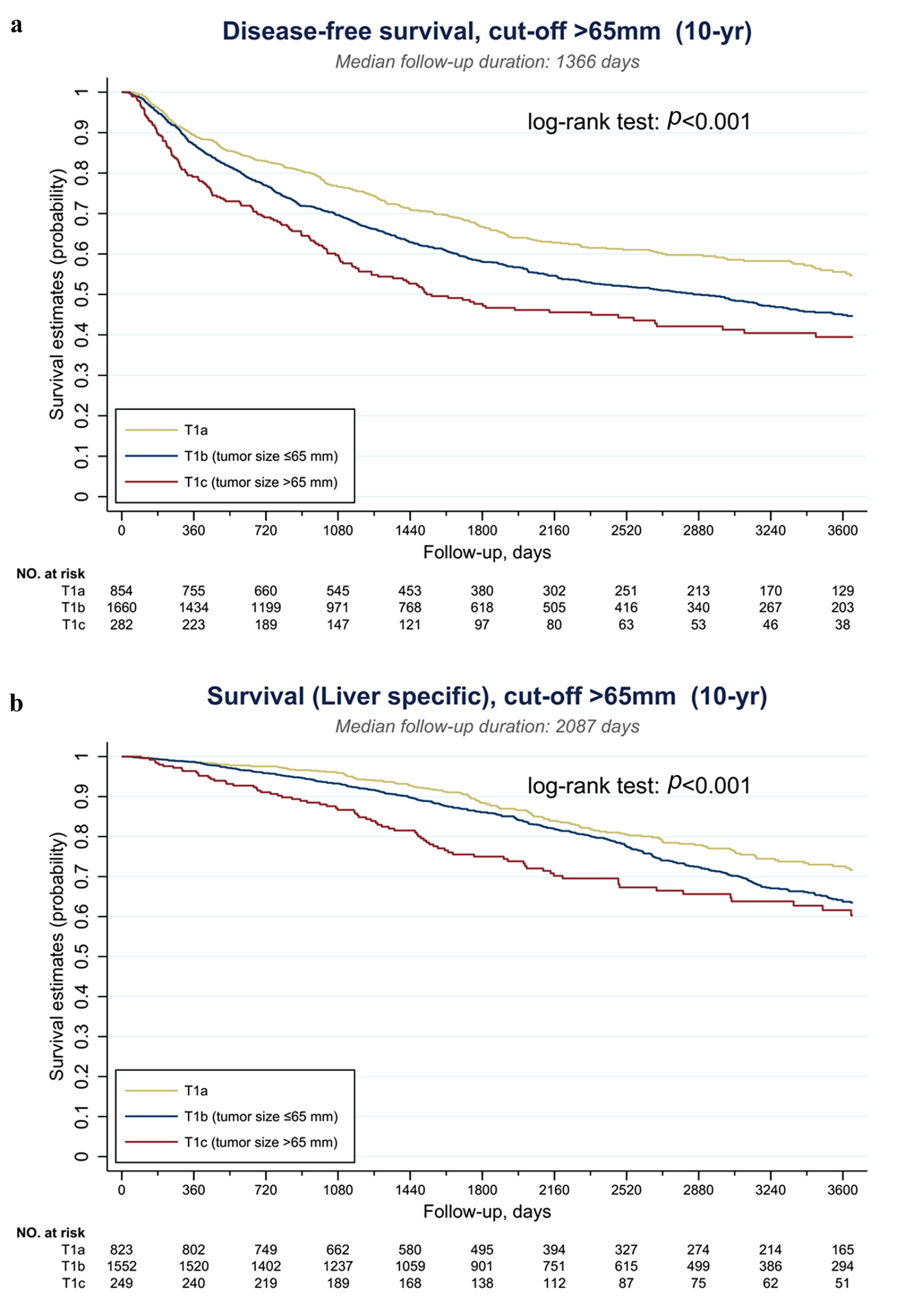
Kaplan–Meier survival curves of subclassified T1 HCC. **a** Disease-free survival (DFS) and **b** liver-specific overall survival (OS)

The serial Kaplan–Meier DFS and liver-specific OS curves of T1cN0M0 and T2N0M0 tumors were demonstrated in Supplementary Figs. S1 and S2, respectively. As shown in Fig. S1, when the cutoff was set below 60 mm, the DFS of T1c tumors was still significantly better than that of T2 tumors (all *p* < 0.05). This survival benefit was obliterated when the cutoff was set at 70 mm (*p* = 0.055). Similarly, the liver-specific OS was significantly longer for T1c tumors when the cutoff was below 60 mm (all *p* < 0.05). This advantage was not observed when the cutoff was set at 70 mm (*p* = 0.099) (Supplementary Fig. S2). To obtain an optimal subclassification for T1 HCC, the cutoff value was further examined at 65 mm and the results are shown in Fig. 2. When T1c was designated as tumors > 65 mm, or > 6.5 cm, the DFS and liver specific-OS were not statistically different from those of T2 tumors (*p* = 0.062 and 0.072, respectively). The optimal cutoff value was thus set at 65 mm, and T1 HCC was further subclassified as T1a: solitary tumor ≤ 2 cm with or without vascular invasion, T1b: solitary tumor > 2 cm but ≤ 6.5 cm without vascular invasion, and T1c: solitary tumor > 6.5 cm without vascular invasion. The Kaplan–Meier DFS and liver-specific OS curves of this new subclassification are illustrated in Fig. 3.

### Clinical characteristics of T1c HCC

The newly proposed T1c subgroup was further analyzed and compared with those T1 HCC ≤ 6.5 cm (T1a/b). As shown in Table 2, around 10% (*n* = 297) of T1 HCC was categorized as T1c. Their mean age of diagnosis was similar to its smaller counterpart, but there were more patients detected at a younger age (≤ 40 years). Of notice, while the incidence of HBV infection was similar between the two groups, there was significantly less HCV-related HCC in T1c group (7.4% vs. 26.6%, *p* < 0.001). There was also much more Child B cirrhosis in T1c HCC (3.2% vs*.* 0.9%, *p* < 0.018). These larger tumors had significantly higher alpha-fetoprotein, platelet count, aspartate aminotransferase (AST), and neutrophil-to-lymphocyte ratio (NLR) but lower hemoglobin, indocyanine green retention test at 15 min (ICG-15) and platelet-to lymphocyte-ratio (PLR). Although albumin and bilirubin levels were similar between the two groups, the derivative ALBI grades were significantly higher in T1c HCC. The nutritional status in terms of prognostic nutritional index (PNI) was also poorer in T1c HCC.

**Table 2:** Baseline demographic features of T1 hepatocellular carcinoma regarding tumor size, *n* (%), mean (SD), *n* = 2876

Variables	Total *n* = 2876	T1 ≦ 6.5 cm (T1a/b) *n* = 2579	T1 > 6.5 cm (T1c) *n* = 297	*p* value
Number^a^	2876 (100%)	2579 (89.7%)	297 (10.3%)	
Gender				
Female	704 (24.5%)	638 (24.7%)	66 (22.2%)	0.34
Male	2172 (75.5%)	1941 (75.3%)	231 (77.8%)	
Age	59.0 (11.2)	58.9 (11.0)	59.2 (13.1)	0.71
≤ 20	2 (0.1%)	1 (0.0%)	1 (0.3%)	0.015
21–40	210 (7.3%)	179 (6.9%)	31 (10.4%)	
41–60	1234 (42.9%)	1123 (43.5%)	111 (37.4%)	
≥ 61	1430 (49.7%)	1276 (49.5%)	154 (51.9%)	
Diabetes				
Yes	717 (24.9%)	651 (25.2%)	66 (22.2%)	0.25
Hypertension				
Yes	1005 (34.9%)	887 (34.4%)	118 (39.7%)	0.068
Hepatitis				**< 0.001**
HBV				
Positive	1385 (48.2%)	1255 (48.7%)	130 (43.8%)	
HCV				
Positive	708 (24.6%)	686 (26.6%)	22 (7.4%)	
HBV + HCV				
Positive	142 (4.9%)	126 (4.9%)	16 (5.4%)	
Cigarette smoking				
Yes	298 (10.4%)	274 (10.6%)	24 (8.1%)	0.17
Alcohol				
Yes	255 (8.9%)	229 (8.9%)	26 (8.8%)	0.94
Betel nut				
Yes	79 (2.7%)	69 (2.7%)	10 (3.4%)	0.49
Child–Turcot–Pugh classification				
A	1464 (98.9%)	1342 (99.1%)	122 (96.8%)	0.018
B	16 (1.1%)	12 (0.9%)	4 (3.2%)	
Cirrhosis				
No cirrhosis	778 (51.0%)	678 (48.4%)	100 (79.4%)	< 0.001
Cirrhosis	748 (49.0%)	722 (51.6%)	26 (20.6%)	
Tumor size (Mm)	35.9 (26.7)	28.6 (13.3)	99.3 (29.6)	< 0.001
Medications				
Anti-HCV/HBV therapy	279 (9.7%)	274 (10.6%)	5 (1.7%)	< 0.001
Metformin	186 (6.5%)	174 (6.7%)	12 (4.0%)	0.073
Aspirin	126 (4.4%)	112 (4.3%)	14 (4.7%)	0.77
Biochemical profiles				
Alpha-fetoprotein (ng/mL)	2471.8 (56,299.0)	745.9 (14,201.9)	18,443.7 (174,587.8)	< 0.001
ICG-15 (%)	9.4 (8.3)	9.6 (8.3)	8.3 (8.6)	0.028
Albumin	4.1 (0.5)	4.1 (0.5)	4.0 (0.6)	< 0.001
Hb	13.6 (1.9)	13.7 (1.9)	13.0 (2.1)	< 0.001
Platelet	175.0 (68.8)	169.5 (64.7)	220.0 (83.4)	< 0.001
INR	1.1 (0.1)	1.1 (0.1)	1.1 (0.1)	0.26
AST	57.4 (97.2)	54.7 (92.7)	81.0 (128.0)	< 0.001
ALT	61.9 (98.7)	61.0 (98.7)	70.0 (98.8)	0.15
Total bilirubin	0.9 (0.6)	0.9 (0.5)	0.9 (0.8)	0.57
ALBI grade				
Grade 1	1,629 (69.9%)	1459 (70.8%)	170 (62.7%)	< 0.001
Grade 2	676 (29.0%)	584 (28.3%)	92 (33.9%)	
Grade 3	27 (1.2%)	18 (0.9%)	9 (3.3%)	
PNI				
Normal	1005 (49.0%)	904 (50.3%)	101 (39.8%)	< 0.001
Mild	531 (25.9%)	463 (25.8%)	68 (26.8%)	
Mod to severe	325 (15.9%)	278 (15.5%)	47 (18.5%)	
Serious	189 (9.2%)	151 (8.4%)	38 (15.0%)	
NLR	4.0 (5.4)	3.8 (4.9)	5.4 (8.1)	< 0.001
PLR	17.3 (6.9)	17.6 (7.1)	14.9 (4.8)	< 0.001

^a^Number excluded surgical mortality (30-day mortality)

*ALBI* albumin–bilirubin grade, *ALT* alanine aminotransferase, *AST* aspartate aminotransferase, *Hb* hemoglobin, *HBV* hepatitis B virus, *HCV* hepatitis C virus, *ICG-15* indocyanine green retention test at 15 min, *INR* international normalized ratio, *NLR* neutrophil-to-lymphocyte ratio, *PLR* platelet-to-lymphocyte ratio, *PNI* prognostic nutritional index, *SD* standard deviation

### Surgical outcome and long-term survival of T1 HCC after liver resection

Table 3 summarizes the surgical and oncological outcome of T1 HCC categorized by our new subclassification. The surgical, or 30-day, mortality rate was comparable between the two groups. The median follow-up time was 67.8 months in T1a/b group and 60.5 months in T1c group. More than 50% of T1c developed tumor recurrence after surgery, compared to only 40% in T1a/b (*p* < 0.001). More than 40% of T1c patients were dead at the end of follow-up, with 25.3% attributed to liver cause. On the other hand, only 27% of T1a/b patients were dead during follow-up (*p* < 0.001). As for the pattern of tumor recurrence, local recurrence remained the most common pattern in T1c HCC, but there was also more regional, combined, or distant recurrence in this group (*p* < 0.001). The 1-, 3-, 5-, and 10-year DFS rates were significantly worse in the T1c group (79.1%, 58.9%, 49.6%, and 46.1%, respectively, in T1c and 87.8%, 72.8%, 64.7%, and 58.8%, respectively, in T1a/b, all *p* < 0.001). Similarly, the 1-, 3-, 5-, and 10-year liver-specific survival rates were remarkably lower in T1c than in T1a/b (96.4%, 87.1%, 77.5%, and 69.9%, respectively, in T1c and 98.6%, 94.4%, 88.8%, and 79.2%, respectively, in T1a/b, all *p* < 0.01).

**Table 3:** Surgical and oncological outcome of patients with T1 HCC

	T1 HCC ≦ 6.5 cm (T1a/b) *n* = 2593	T1 HCC > 6.5 cm (T1c) *n* = 300	*p* value
Surgical mortality (30 days)	14 (0.5%)	3 (1.0%)	0.32
Recurrence status			
No recurrence	1480 (57.4%)	130 (43.8%)	< 0.001
Recurrence	1035 (40.1%)	152 (51.2%)	
Never disease free	64 (2.5%)	15 (5.1%)	
Follow-up times (months) [Median (IQR)]	67.8 (39.5–105.4)	60.5 (33.0–100)	0.015
Final status			
Alive	1880 (72.9%)	174 (58.6%)	< 0.001
Death—liver cause	495 (19.2%)	75 (25.3%)	
Death—other cause	204 (7.9%)	48 (16.2%)	

	T1 HCC ≦ 6.5 cm *n* = 2515	T1 HCC > 6.5 cm *n* = 282	*p* value
Recurrence pattern			
Local^a^	710 (28.3%)	90 (32.0%)	< 0.001
Regional^b^	53 (2.1%)	14 (5.0%)	
Combined^c^	18 (0.7%)	7 (2.5%)	
Distant	27 (1.1%)	10 (3.6%)	
Death without recurrence	225 (9.0%)	30 (10.7%)	
Disease free survival			
1-year DFS rate	2207 (87.8%)	223 (79.1%)	< 0.001
3-year DFS rate	1832 (72.8%)	166 (58.9%)	< 0.001
5-year DFS rate	1627 (64.7%)	140 (49.6%)	< 0.001
10-year DFS rate	1480 (58.8%)	130 (46.1%)	< 0.001

	T1 HCC ≦ 6.5 cm *N* = 2375	T1 HCC > 6.5 cm *n* = 249	*p* value
Liver-specific survival			
1-year survival rate	2342 (98.6%)	240 (96.4%)	0.008
3-year survival rate	2242 (94.4%)	217 (87.1%)	< 0.001
5-year survival rate	2110 (88.8%)	193 (77.5%)	< 0.001
10-year survival rate	1880 (79.2%)	174 (69.9%)	< 0.001

*DFS* disease-free survival, *HCC* hepatocellular carcinoma, *IQR* interquartile range

^a^Local recurrence include resection margin/remnant liver or trocar site

^b^Regional recurrence include adjacent organs/regional LNs, or both

^c^Combined recurrence include local and regional recurrence

### Multivariate analysis of risk factors for tumor recurrence and mortality in T1 HCC

In addition to tumor size > 6.5 cm, age ≥ 65 years, histological cirrhosis, diabetes mellitus, chronic HCV infection, hemoglobin ≤ 10 g/dL, and albumin ≤ 3.5 g/dL were found to be related to tumor recurrence after liver resection for T1 HCC (all *p* < 0.05). HBV infection and administration of antiviral therapy, on the other hand, were associated with less tumor recurrence after surgery. Cox regression multivariate analysis further demonstrated that age ≥ 65 years, histological cirrhosis, and hemoglobin ≤ 10 g/dL, in addition to tumor size > 6.5 cm, were independent prognostic factors for HCC recurrence after surgery (Table 4). Tumors larger than 6.5 cm were 1.61-fold more likely to develop tumor relapse than those smaller than 6.5 cm. Likewise, tumor size > 6.5 cm, histological cirrhosis, and hemoglobin ≤ 10 g/dL were independent predicting factors for liver-specific mortality after liver resection for T1 HCC (all *p* < 0.05) (Table 5). Tumors larger than 6.5 cm had a 1.74-fold risk of liver-specific death after liver resection.

**Table 4:** Univariate and multivariate analyses of risks factors for tumor recurrence after hepatectomy for AJCC 8 T1 hepatocellular carcinoma

Variables	Univariate		Multivariate	
	HR (95% CI)	*p* value	HR (95% CI)	*p* value
Gender				
Female	1 (reference)			
Male	0.99 (0.87–1.13)	0.922	1.30 (1.00–1.68)	0.050
Age				
< 65 y/o	1 (reference)			
≥ 65 y/o	1.57 (1.40–1.76)	< 0.001	1.26 (1.01–1.58)	0.044*
Tumor size				
≤ 6.5 cm	1 (reference)			
> 6.5 cm	1.45 (1.21–1.73)	< 0.001	1.61 (1.13–2.28)	0.008**
Cirrhosis				
No	1 (reference)			
Yes	1.54 (1.29–1.85)	< 0.001	1.58 (1.26–1.98)	< 0.001***
Diabetes mellitus				
No	1 (reference)			
Yes	1.17 (1.03–1.33)	0.016	1.00 (0.79–1.26)	0.975
Hypertension				
No	1 (reference)			
Yes	1.06 (0.94–1.19)	0.323		
Alcohol				
No	1 (reference)			
Yes	0.90 (0.72–1.12)	0.342		
HBs Ag				
Negative	1 (reference)		0.82 (0.63–1.06)	0.132
Positive	0.70 (0.63–0.79)	< 0.001		
Hepatitis C virus				
Negative	1 (reference)			
Positive	1.41 (1.25–1.58)	< 0.001	1.24 (0.95–1.61)	0.110
Hemoglobin				
> 10 (g/dL)	1 (reference)			
≤ 10	1.66 (1.29–2.12)	< 0.001	1.78 (1.15–2.75)	0.010**
INR				
≤ 1.4	1 (reference)			
> 1.4	0.90 (0.41–1.97)	0.791		
Albumin				
> 3.5 (g/dL)	1 (reference)			
≤ 3.5	1.50 (1.27–1.77)	< 0.001	1.02 (0.71–1.47)	0.896
α-Fetoprotein				
≤ 400 (ng/mL)	1 (reference)			
> 400	1.07 (0.88–1.29)	0.514	1.11 (0.80–1.53)	0.527
Antiviral therapy in HBV or HCV infection				
No	1 (reference)			
Yes	0.68 (0.55–0.86)	0.001	0.74 (0.52–1.06)	0.102
NLR (continuous variables)	1.00 (0.99–1.01)	0.986		

The covariates with significant statistics and with important clinical implications were put into the multivariate Cox regressions

*CI* confidence interval, *HBs Ag* hepatitis B surface antigen, *HBV* hepatitis B virus, *HCV* hepatitis C virus, *HR* hazard ration, *INR* international normalized ratio

**Table 5:** Univariate and multivariate analyses of risks factors for liver-specific mortality after hepatectomy for AJCC 8 T1 hepatocellular carcinoma

Variables	Univariate		Multivariate (selected)	
	HR (95% CI)	*p* value	HR (95% CI)	*p* value
Gender				
Female	1 (reference)			
Male	0.92 (0.76–1.11)	0.368	1.04 (0.70–1.55)	0.845
Age				
< 65 y/o	1 (reference)			
≥ 65 y/o	1.97 (1.67–2.33)	< 0.001	1.33 (0.93–1.91)	0.119
Tumor size				
≤ 6.5 cm	1 (reference)			
> 6.5 cm	1.51 (1.17–1.95)	0.001	1.74 (1.02–2.96)	0.043*
Cirrhosis				
No	1 (reference)			
Yes	1.56 (1.17–2.10)	0.003	1.71 (1.19–2.45)	0.004**
Diabetes mellitus				
No	1 (reference)			
Yes	1.34 (1.11–1.61)	0.002	0.86 (0.58–1.26)	0.428
Alcohol				
No	1 (reference)			
Yes	1.13 (0.81–1.57)	0.459		
HBs Ag				
Negative	1 (reference)			
Positive	0.67 (0.57–0.79)	< 0.001	0.86 (0.57–1.30)	0.479
Hepatitis C virus negative	1 (reference)			
Positive	1.36 (1.15–1.62)	< 0.001	1.25 (0.82–1.89)	0.301
Hemoglobin				
> 10 (g/dL)	1 (reference)			
≤ 10	2.30 (1.64–3.23)	< 0.001	2.96 (1.69–5.18)	< 0.001***
INR				
≤ 1.4	1 (reference)			
> 1.4	0.95 (0.35–2.60)	0.926		
Albumin				
> 3.5 (g/dL)	1 (reference)			
≤ 3.5	1.64 (1.30–2.08)	< 0.001	1.24 (0.75–2.06)	0.405
α-Fetoprotein				
≤ 400 (ng/mL))	1 (reference)			
> 400	0.99 (0.75–1.31)	0.932	1.06 (0.63–1.79)	0.817
Antiviral therapy in HBV or HCV infection				
No	1 (reference)		0.70 (0.39 1.27)	0.240
Yes	0.71 (0.51–0.99)	0.043		
NLR (continuous variables)	0.99 (0.98–1.01)	0.425		

The covariates with significant statistics and with important clinical implications were put into the multivariate Cox regressions

*CI* confidence interval, *HBs Ag* hepatitis B surface antigen, *HBV* hepatitis B virus, *HCV* hepatitis C virus, *HR* hazard ration, *INR* international normalized ratio

To further confirm the validity of our newly proposed T1 subclassfication, sensitivity tests comprising different combinations of variables were conducted and the results are summarized in Table 6. Variables including gender, age, diabetes mellitus (DM), HBV, HCV, NLR, hemoglobin, albumin, antiviral therapy, liver cirrhosis, and ALBI grade, in addition to tumor size > 6.5 cm, were incorporated into 5 different models. The adjusted hazard ratio (aHR) of tumor size > 6.5 cm for either tumor recurrence or liver-specific mortality in the respective models was analyzed and computed. When comparing with smaller tumors, tumors lager than 6.5 cm were significantly more likely to develop both tumor recurrence and liver-specific mortality across all models, with aHR ranging between 1.46 and 1.67 for recurrence and 1.54 and 1.81 for liver-specific mortality.

**Table 6:** Sensitivity tests

Models	Tumor recurrence		Liver-specific mortality	
	aHR^b^ (95% CI)	*p* value	aHR^b^ (95% CI)	*p* value
Model 1: Tumor size^a^, gender, age, DM, HBV, HCV, NLR	1.46 (1.21–1.77)	< 0.001	1.54 (1.18–2.01)	0.001
Model 2: Tumor size^a^, cirrhosis, AFP, Hb	1.67 (1.20–2.32)	0.002	1.80 (1.09–2.99)	0.022
Model 3: Tumor size^a^, cirrhosis, AFP, Hb, gender, age, DM, HBV, HCV, anti-HBV/HCV therapy	1.61 (1.15–2.27)	0.006	1.81 (1.08–3.03)	0.023
Model 4: Tumor size^a^, cirrhosis, AFP, Hb, gender, age, DM, HBV, HCV, anti-HBV/HCV therapy, ALBI	1.61 (1.13–2.28)	0.008	1.74 (1.02–2.96)	0.043
Model 5: Tumor size^a^, cirrhosis, AFP, Hb, gender, age, DM, HBV, HCV, anti-HBV/HCV therapy, albumin	1.60 (1.13–2.26)	0.009	1.73 (1.02–2.94)	0.042

*ALBI* albumin–bilirubin grade, *AFP* alpha-fetoprotein, *aHR* adjusted hazard ration, *CI* confidence interval, *DM* diabetes mellitus, *Hb* hemoglobin, *HBV* hepatitis B virus, *HCV* hepatitis C virus, *INR* international normalized ratio, *NLR* neutrophil-to-lymphocyte ratio

^a^Tumor size > 6.5 cm vs. ≤ 6.5 cm

^b^aHR of tumor size > 6.5 cm as computed in different models

## Discussion

According to the 8th edition of AJCC TNM staging system for HCC, solitary small tumor (< 5 cm) without vascular invasion and solitary huge tumor (> 10 cm) without vascular invasion were all categorized as T1 lesions [9, 10]. As a result, the treatment recommendation and prognosis were deemed to be similar between these two entities. Nevertheless, our recent study demonstrated that in stage I HCC after liver resection, tumors larger than 10 cm had significantly higher risks of tumor recurrence and death than those smaller than 10 cm [15]. Single HCC > 5 cm without vascular invasion, in another study, was found to have a survival rate inferior to that of HCC < 5 cm [12]. There was even another report suggesting that single HCC should be assigned into three different groups according to the tumor size (≤ 5 cm, > 5 and ≤ 8 cm, and > 8 cm, respectively) [13]. Patients with larger HCC, as a result, did not have survival outcome comparable to those with smaller tumors as expected. A recent study further proposed that, due to their similar survivals, the T1b lesions should be integrated with T2 lesions to obtain a modified TNM staging [11]. These studies all indicated that tumor size did significantly impact the outcome of solitary HCC and should be considered in the conventional staging systems. In other words, there should be a subclassification within T1 stage to precisely predict patient outcome. The exact cutoff values, however, are still controversial among different literatures. The current study, by examining one of the largest and most comprehensive clinical databases worldwide, discovered that solitary HCC > 6.5 cm without vascular invasion had a DFS and liver specific-OS similar to those of T2 tumors. Hence, we proposed that T1 HCC can be further classified to accommodate a T1c subcategory: solitary tumor > 6.5 cm without vascular invasion. To further corroborate our findings, we performed Cox regression multivariate analysis and found that tumor size > 6.5 cm was one of the independent prognostic factors for tumor recurrence and liver-specific mortality after liver resection for T1 HCC. By conducting sensitivity tests consisting of different models, we again demonstrated that tumor size did influence the outcome of T1 HCC. All statistics in the sensitivity tests showed the same trend that patients with HCC larger than 6.5 cm had higher risks of recurrence and liver-specific death. This cutoff, coincidentally, corresponded to the criteria suggested by the University of California San Francisco (UCSF) that a solitary HCC larger than 6.5 cm without vascular invasion had significantly worse survivals after orthotopic liver transplantation [22, 23]. Therefore, we believe tumor size > 6.5 cm is an important prognostic factor in T1 HCC and should be considered in the TNM staging system.

Unlike a previous study which claimed that tumor size was not a prominent prognostic indicator in nonvascular invading solitary HCC receiving liver resection, the current study discovered that tumor size > 6.5 cm was indeed a significant prognostic factor among T1 HCC patients undergoing surgery [24]. The inconsistent results may be explained by the different study designs. In the current study, we incrementally divided T1b tumors into two groups and tried to find out a cutoff which could differentiate the new subcategory from the remaining T1 tumors. The resulting T1c subgroup had a survival, not only significantly shorter than the T1a/b tumors, but similarly poor with the T2 tumors. In contrast, the study conducted by Yang et al*.* divided patients into three groups (≤ 30 mm, 31‐50 mm, and > 50 mm) based on tumor size limitations between radiofrequency ablation and liver transplantation [24]. They compared the survival outcome of larger tumors (31‐50 mm or > 50 mm) against those of smaller ones (≤ 30 mm). The different cutoffs and analyses may lead to different conclusions. The disparate AJCC versions also rendered these results incomparable. The current study, by adopting the newest 8th edition of AJCC TNM staging system for HCC, is readily available to be applied in the real world clinical practice. Due to inferior survival outcome, the treatment strategy and surveillance protocol should be modified for this subgroup of patients. Adjuvant treatment with either transarterial chemoembolization (TACE) or systemic therapy (tyrosine kinase inhibitors or immune checkpoint inhibitors) could be considered for this subset of patients. Further prospective clinical trials are warranted to establish a more effective treatment protocol for these patients.

In addition to worse survivals, the current study also discovered that T1 HCC > 6.5 cm had less HCV infection and cirrhosis. There were more patients diagnosed at a younger age, too. Since younger HCC patients have been demonstrated to have lower rates of HCV infection and cirrhosis, it may explain the demographic disparities observed [25]. However, it is also likely that the carcinogenesis of T1c HCC is different from that of T1a/b tumors. The non-viral cause, such as fatty liver diseases, might have contributory roles in the pathogenesis of these large tumors. This speculation can be supported by our finding that non-viral cause accounted for more than 54% of T1c HCC in the present study, as compared to only 30% of T1a/b tumors (*p* < 0.001). Further studies are warranted to explore the causal relationships between these associations.

Despite remarkable findings, the current study still has several limitations. First, the current study was generated from the hospital-based database and cancer registry, more descriptive features, such as performance status, postoperative complications, and pathologic details; for example, hepatitis activity index, margin status, and histological grade, were inaccessible. The analysis of these variables was thus lacking. Second, since some T2 lesions, for example, bilobar tumors or more than 3 tumors, may not undergo surgery, the surgical survivals obtained herein may not fully represent the outcome associated with all T2 lesions. This was why we did not propose to upgrade our new “T1c” subcategory into T2 stage (Supplementary Fig. S3). We only intended to find out an optimal size cutoff within the T1 stage to differentiate those patients at risk. As a result, further studies are warranted to examine whether T1c stage should be integrated into T2 stage. Third, as mentioned above, the current study failed to suggest adequate treatment strategy for T1c lesions. Further well-designed prospective studies targeting at this subset of patients are thus necessary to establish appropriate treatment guidelines. Next, although the potential recall bias could be avoided by prospectively registering the daily clinical data into the CGRD, referral bias was in the meanwhile inevitable, since the CGMHs are the largest tertiary care center in Taiwan [26, 27]. Last but not the least, since the present study was based primarily on data from a single country, the patient population as a result would be rather uniform. The lack of an external validation cohort consisted of different ethnic groups, therefore, would be another drawback of the current study. It would be more convincing if the data can be confirmed by HCC data sets from countries with a more diverse population. Further studies incorporating external validation cohorts are necessary to approve our findings.

## Conclusions

Our CGRD-based study demonstrated that tumor size would significantly impact the survival outcome of T1 HCC after surgery. Solitary tumor > 6.5 cm without vascular invasion, after serial analysis, was found to have a survival similar to that of T2 HCC. As a result, we proposed a subclassification within T1 HCC, T1c: solitary tumor > 6.5 cm without vascular invasion, to further stratify those patients at risk. Due to significantly higher risks of recurrence and death, adjuvant treatment should be considered for this subset of T1 HCC. Further studies are mandatory to validate our findings.

## Supplementary Information

Below is the link to the electronic supplementary material.

Supplementary Figure S1. Kaplan-Meier disease-free survival (DFS) curves of T1c and T2 HCC, illustrated by different cutoffs (TIF 398 KB)

Supplementary Figure S2. Kaplan-Meier overall survival (OS) curves of T1c and T2 HCC, illustrated by different cutoffs. (TIF 404 KB)

Supplementary Figure S3. Kaplan-Meier disease-free survival (DFS) curve and overall survival (OS) curve of modified T1 and T2 HCC. Modified T1: T1a, solitary tumor ≤ 2 cm with or without vascular invasion, or T1b, solitary tumor > 2 cm but ≤ 6.5 cm without vascular invasion; Modified T2: solitary tumor > 6.5 cm without vascular invasion, solitary tumor > 2 cm with vascular invasion, or multiple tumors none greater than 5 cm in diameter (TIF 339 KB)

## Data Availability

All data generated or analyzed during the study are included in this published article. Raw data may be requested from the authors with the permission of the institution.

## Abbreviations

AFP: α-Fetoprotein
aHR: Adjusted HR
AJCC: American Joint Committee on Cancer
ALBI: Albumin–bilirubin
ALT: Alanine aminotransferase
AST: Aspartate aminotransferase
CGMH: Chang Gung memorial hospital
CGRD: Chang Gung Research Database
CI: Confidence interval
DFS: Disease-free survival
HBV: Hepatitis B virus
HCV: Hepatitis C virus
HCC: Hepatocellular carcinoma
HR: Hazard ratio
ICD-9-CM: International classification of diseases, 9th revision, clinical modification
ICD-10-CM: International classification of diseases, 10th revision, clinical modification
ICD-O-3: International classification of diseases for oncology, 3rd edition
ICG-15: Indocyanine green retention test at 15 min
INR: International normalized ratio
IRB: Institutional Review Board
NLR: Neutrophil-to-lymphocyte ratio
OS: Overall survival
PLR: Platelet-to-lymphocyte ratio
PNI: Prognostic nutritional index
SD: Standard deviation
TACE: Transarterial chemoembolization
TNM: Tumor/node/metastasis
UCSF: University of California San Francisco

## References

[1] Torre LA, Bray F, Siegel RL, Ferlay J, Lortet-Tieulent J, Jemal A (2015). Global cancer statistics, 2012. CA Cancer J Clin.

[2] Bray F, Ferlay J, Soerjomataram I, Siegel RL, Torre LA, Jemal A (2018). Global cancer statistics 2018: GLOBOCAN estimates of incidence and mortality worldwide for 36 cancers in 185 countries. CA Cancer J Clin.

[3] Llovet JM, Kelley RK, Villanueva A, Singal AG, Pikarsky E, Roayaie S (2021). Hepatocellular carcinoma. Nat Rev Dis Prim.

[4] Sung H, Ferlay J, Siegel RL, Laversanne M, Soerjomataram I, Jemal A (2021). Global cancer statistics 2020: GLOBOCAN estimates of incidence and mortality worldwide for 36 cancers in 185 countries. CA Cancer J Clin.

[5] Department of Health ROC (2021). Statistics of cause of death in Taiwan 2020.

[6] Forner A, Llovet JM, Bruix J (2012). Hepatocellular carcinoma. Lancet (London, England).

[7] Edge SB, Byrd DR, Carducci MA, Compton CC, Fritz A, Greene F (2010). AJCC cancer staging manual.

[8] Greene FL, Sobin LH (2009). A worldwide approach to the TNM staging system: collaborative efforts of the AJCC and UICC. J Surg Oncol.

[9] Edge SB, Byrd DR, Carducci MA, Compton CC, Fritz A, Greene F (2017). AJCC cancer staging manual.

[10] Amin MB, Greene FL, Edge SB, Compton CC, Gershenwald JE, Brookland RK (2017). The eighth edition AJCC cancer staging manual continuing to build a bridge from a population-based to a more “personalized” approach to cancer staging. CA Cancer J Clin.

[11] Abdel-Rahman O (2018). Assessment of the discriminating value of the 8th AJCC stage grouping for hepatocellular carcinoma. HPB Off J Int Hepato Pancreato Biliary Assoc.

[12] Fang KC, Kao WY, Su CW, Chen PC, Lee PC, Huang YH (2018). The prognosis of single large hepatocellular carcinoma was distinct from barcelona clinic liver cancer stage A or B: the role of albumin-bilirubin grade. Liver Cancer.

[13] Zhong JH, Pan LH, Wang YY, Cucchetti A, Yang T, You XM (2017). Optimizing stage of single large hepatocellular carcinoma: a study with subgroup analysis by tumor diameter. Medicine.

[14] Shindoh J, Kobayashi Y, Kawamura Y, Akuta N, Kobayashi M, Suzuki Y (2020). Microvascular invasion and a size cutoff value of 2 cm predict long-term oncological outcome in multiple hepatocellular carcinoma: reappraisal of the American joint committee on cancer staging system and validation using the surveillance, epidemiology, and end-results database. Liver Cancer.

[15] Lee CW, Yu MC, Wang CC, Lee WC, Tsai HI, Kuan FC (2021). Liver resection for hepatocellular carcinoma larger than 10 cm: a multi-institution long-term observational study. World J Gastrointest Surg.

[16] Shao SC, Chan YY, Kao Yang YH, Lin SJ, Hung MJ, Chien RN (2019). The Chang Gung Research Database-a multi-institutional electronic medical records database for real-world epidemiological studies in Taiwan. Pharmacoepidemiol Drug Saf.

[17] Tsai MS, Lin MH, Lee CP, Yang YH, Chen WC, Chang GH (2017). Chang Gung Research Database: a multi-institutional database consisting of original medical records. Biomed J.

[18] Liu JM, Lin CC, Liu KL, Lin CF, Chen BY, Chen TH (2020). Second-line hormonal therapy for the management of metastatic castration-resistant prostate cancer: a real-world data study using a claims database. Sci Rep.

[19] Chiang CJ, You SL, Chen CJ, Yang YW, Lo WC, Lai MS (2015). Quality assessment and improvement of nationwide cancer registration system in Taiwan: a review. Jpn J Clin Oncol.

[20] Chiang CJ, Wang YW, Lee WC (2019). Taiwan’s nationwide cancer registry system of 40 years: past, present, and future. J Formos Med Assoc.

[21] Berthold MR, Cebron N, Dill F, Gabriel TR, Kötter T, Meinl T (2009). KNIME - the Konstanz information miner: version 2.0 and beyond. SIGKDD Explor Newsl.

[22] Yao FY, Ferrell L, Bass NM, Watson JJ, Bacchetti P, Venook A (2001). Liver transplantation for hepatocellular carcinoma: expansion of the tumor size limits does not adversely impact survival. Hepatology.

[23] Unek T, Karademir S, Arslan NC, Egeli T, Atasoy G, Sagol O (2011). Comparison of Milan and UCSF criteria for liver transplantation to treat hepatocellular carcinoma. World J Gastroenterol.

[24] Yang A, Xiao W, Chen D, Wei X, Huang S, Lin Y (2018). The power of tumor sizes in predicting the survival of solitary hepatocellular carcinoma patients. Cancer Med.

[25] Yeh C-N, Lee W-C, Chen M-F (2003). Hepatic resection and prognosis for patients with hepatocellular carcinoma larger than 10 cm: two decades of experience at Chang Gung memorial hospital. Ann Surg Oncol.

[26] Sedgwick P (2012). What is recall bias?. BMJ.

[27] Sedgwick P (2014). Retrospective cohort studies: advantages and disadvantages. Br Med J.

