# Supplementary figures and images for: A proposal for T1 subclassification in hepatocellular carcinoma: reappraisal of the AJCC 8th edition
Source: Hepatol Int. 2022 Sep 28;16(6):1353–67. doi: 10.1007/s12072-022-10422-8 (PMC9701177; doi:10.1007/s12072-022-10422-8)

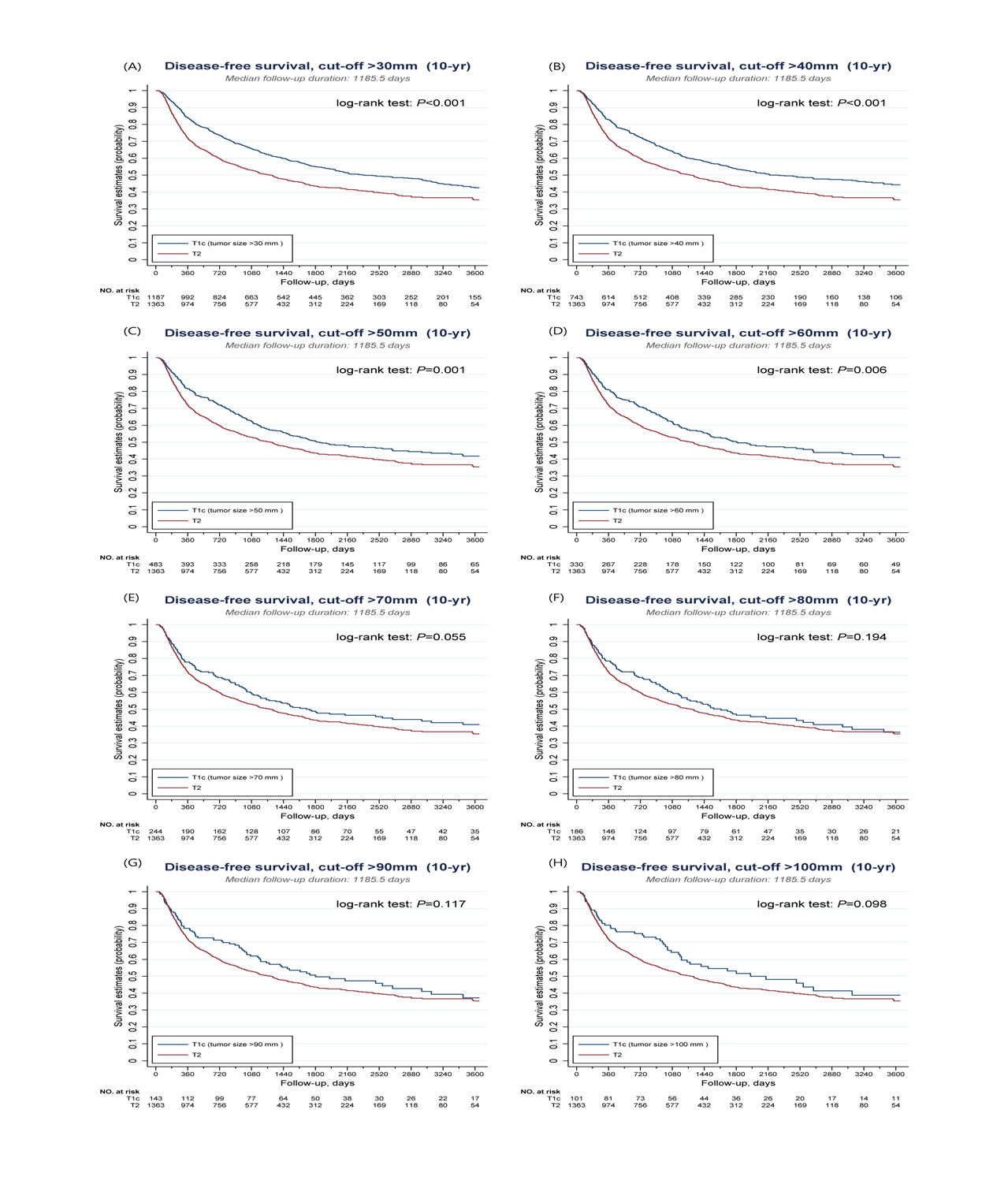

Supplement: Supplementary file 1 — Supplementary Figure S1. Kaplan-Meier disease-free survival (DFS) curves of T1c and T2 HCC, illustrated by different cutoffs (TIF 398 KB) [file 12072_2022_10422_MOESM1_ESM.tif]

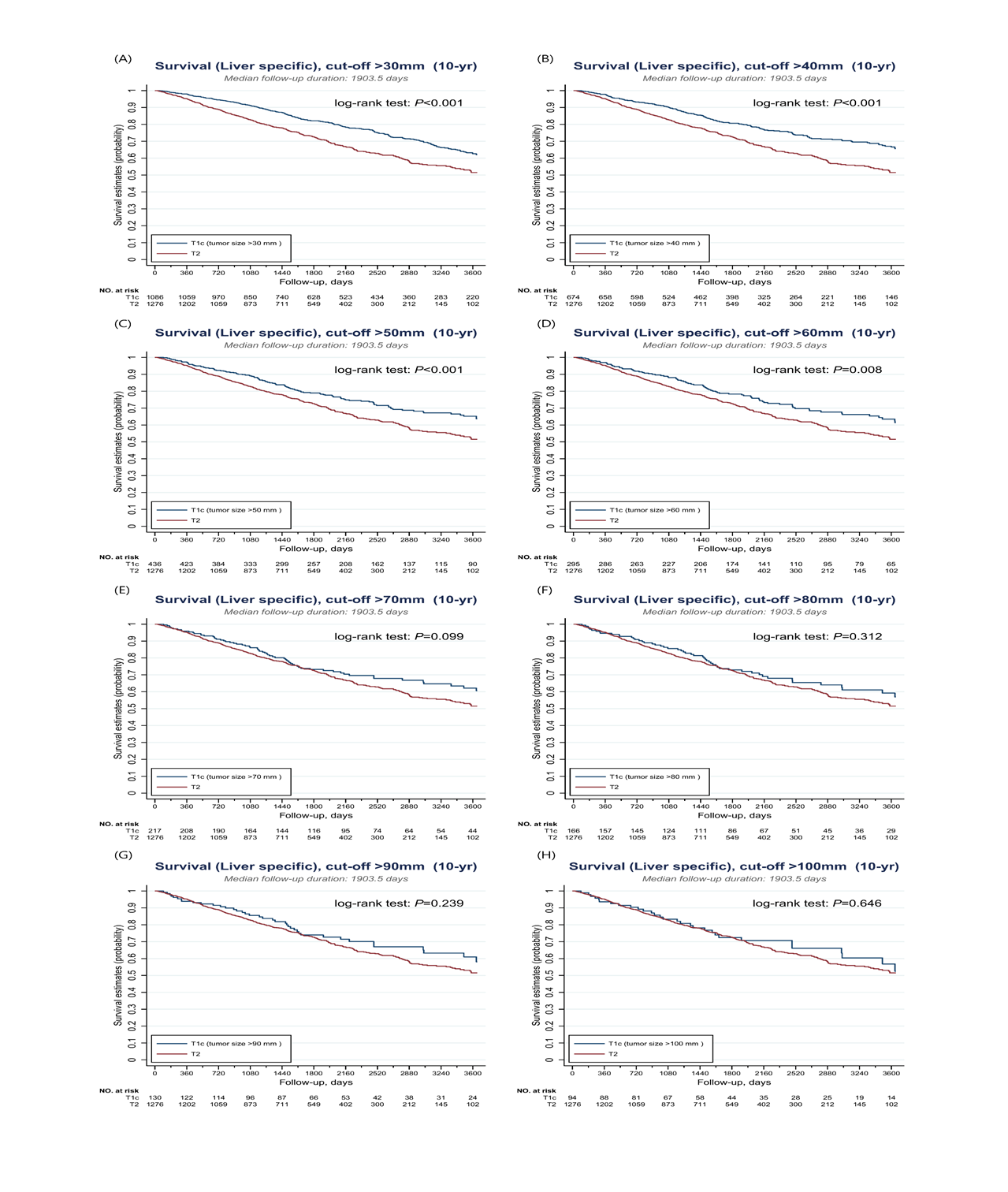

Supplement: Supplementary file 2 — Supplementary Figure S2. Kaplan-Meier overall survival (OS) curves of T1c and T2 HCC, illustrated by different cutoffs. (TIF 404 KB) [file 12072_2022_10422_MOESM2_ESM.tif]

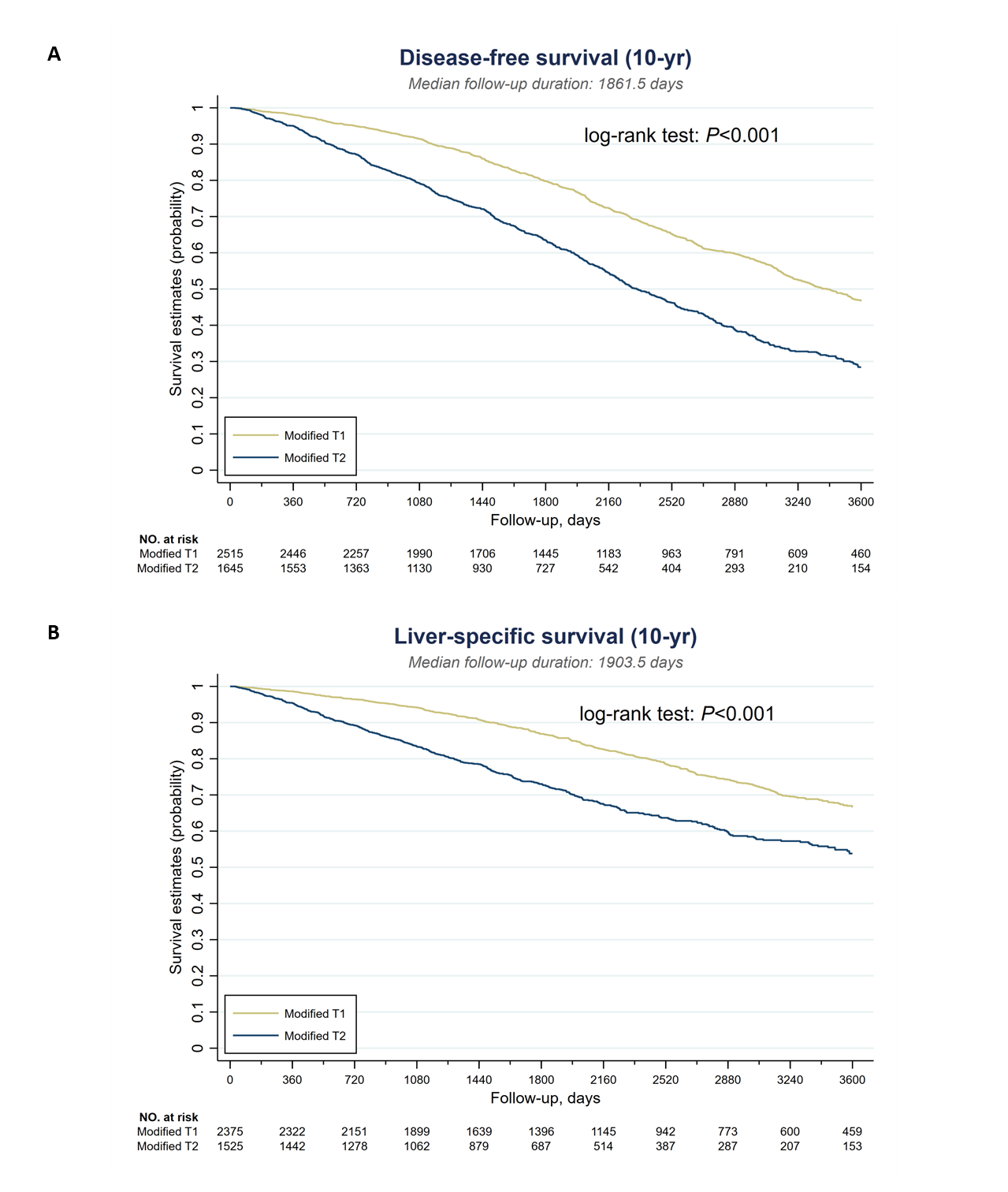

Supplement: Supplementary file 3 — Supplementary Figure S3. Kaplan-Meier disease-free survival (DFS) curve and overall survival (OS) curve of modified T1 and T2 HCC. Modified T1: T1a, solitary tumor ≤ 2 cm with or without vascular invasion, or T1b, solitary tumor > 2 cm but ≤ 6.5 cm without vascular invasion; Modified T2: solitary tumor > 6.5 cm without vascular invasion, solitary tumor > 2 cm with vascular invasion, or multiple tumors none greater than 5 cm in diameter (TIF 339 KB) [file 12072_2022_10422_MOESM3_ESM.tif]
